# Measuring the sustainability of neighborhoods: A systematic literature review

**DOI:** 10.1016/j.isci.2023.105951

**Published:** 2023-01-10

**Authors:** Mahsa Khatibi, Khairul Anwar Mohamed Khaidzir, Sharifah Salwa Syed Mahdzar

**Affiliations:** 1 Department of Architecture, Faculty of Built Environment and Surveying, Universiti Teknologi Malaysia, 81310 Skudai, Johor, Malaysia; 2 Architecture Department, Engineering Faculty, Herat University, Herat, Afghanistan

**Keywords:** Environmental policy, Environmental issues, Urban planning, Research methodology social sciences

## Abstract

Neighborhoods have received worldwide interest in sustainability assessment due to their suitable scale for representing the relationship between the individual and the city. Consequently, this has led to a focus on developing neighborhood sustainability assessment (NSA) systems and, thereby, studying the prominent NSA tools. Alternatively, this study aims to uncover formative concepts shaping the assessment of sustainable neighborhoods based on a systematic review of the empirical work by researchers. The study included a Scopus database search for papers measuring neighborhood sustainability and a literature review of 64 journal articles published between 2019 and 2021. Our results suggest that criteria related to sustainable form and morphology are the most widely measured criteria in the reviewed papers, interconnected with multiple aspects of neighborhood sustainability. The paper contributes to expanding the existing knowledge on neighborhood sustainability evaluation, further adding to the literature on designing sustainable cities and communities and achieving Sustainable Development Goal 11.

## Highlights

• A systematic literature review and analysis of 64 journal articles are conducted
• The frequently measured criteria for NSA are identified using the NVIVO software
• Urban form and morphology are revealed as the widely measured NSA factors
• A tendency to integrate environmental measures with social aspects is noticed

## Graphical abstract

**Figure:**
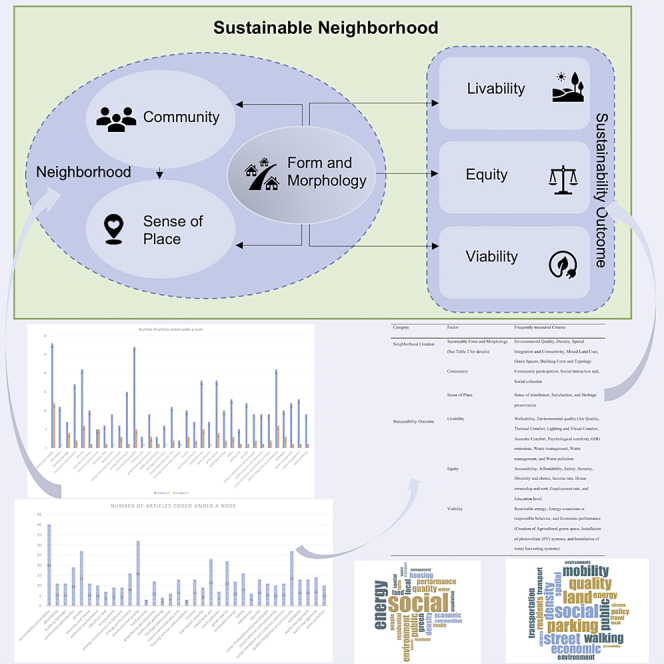

## Introduction

Sustainable development has become a significant challenge in the twenty-first century.^1^ As urban population grows, many practitioners and policymakers recognize the importance of formulating and implementing strategies that lead to sustainable urban development in cities.^2^ The Rio Earth Summit Local Agenda 21, published in 1992, was the first document to address sustainability at the local level. Since then, the concept of a sustainable city has gained considerable political resonance worldwide and is further exemplified by Sustainable Development Goal 11, which focuses on sustainable, resilient, safe, and inclusive cities and communities.^3^

In contrast, there is a growing debate about the various forms of inequality as an obstacle to transition to urban resilience and sustainability. These include inequality due to varying social conditions, lack of access to basic infrastructure such as transportation, and exposure to environmental stresses such as pollution in many contemporary cities.^4^^,^^5^ According to Subramanian et al.^5^ based on Sampson,^6^ the spatial inequalities arise because of the interconnectedness and intertwined nature of social, environmental, and economic outcomes. Therefore, novel tools and theoretical ideas are needed to integrate the multiple and intertwined sustainability dimensions.^5^

The built environment provides a notable context for the comprehensive and integrated implementation of sustainable initiatives, not least the monitoring of rapid transition to sustainability in cities. In principle, the methodologies for promoting and assessing sustainable development through built environment exist at two main scales, i.e., building and urban.^7^^,^^8^ As recent literature points to certain shortcomings and deficiencies in the building-level assessment, there is an increased focus nowadays on sustainability assessment of urban areas, especially the neighborhood sustainability assessment.^7^^,^^8^^,^^9^^,^^10^^,^^11^^,^^12^

There are a variety of descriptions for neighborhoods. Neighborhoods are mostly recalled as "the building blocks of a city" by researchers.^13^^,^^14^^,^^15^^,^^16^^,^^17^ They are also considered a place representing the relationship between the city and the individual.^18^ Following Kallus and Law-yone,^16^ neighborhoods function as components of an urban settlement that aims to bridge the gap between the individual home and the overall urban environment. Despite the extensive research, the definition of neighborhood varies widely concerning its boundaries.^15^

As research on sustainable neighborhoods increased, the development of monitoring tools and assessment methods to evaluate sustainability gained the focus of policymakers and researchers. The building industry and construction market responded to this scenario by developing a variety of neighborhood sustainability assessment (NSA) tools, namely Building Research Establishment Environmental Assessment Method for Communities (BREEAM-C), Leadership in Energy and Environmental Design for Neighborhood Development (LEED-ND), Comprehensive Assessment System for Building Environmental Efficiency for Urban Development (CASBEE-UD), and Green Star Communities. These prominent NSA tools provide the means to define and assess neighborhood sustainability.^19^^,^^20^

Recently, however, many academics and researchers have begun to address the shortcomings of these tools, including a bias toward environmental sustainability, a focus on the ecological parameters of a city, and a lack of consideration of the local context.^1^^,^^7^^,^^11^ Borges et al.^21^ also highlighted the technocratic and rationalist nature of the tools that disregards human priorities and values. To create a more equitable perspective, all three main pillars of sustainability, i.e., social, environmental, and economic, should be adequately considered in formulating sustainable neighborhood strategies and trade-off measures. Indeed, scholars have also emphasized the importance of considering governance, management, culture, and institutional dimensions as critical aspects of neighborhood sustainability.^8^^,^^22^^,^^23^^,^^24^ Therefore, it is vital to understand the concept of a sustainable neighborhood from the perspective of researchers and scholars.

According to Salomaa and Juhola,^25^ based on their article on sustainable transformation, a social phenomenon (such as a neighborhood) is always conceptualized using different methods, which is not a problem. However, it is of concern that despite rigorous advocacy for sustainable transformation of societies and communities, there is still a lack of clarity on this issue and a lack of means to assess it. Fearing that the term "sustainability" and "transformation" may become a mere rhetorical tool of discourse, a deeper understanding of its underlying concepts and the way it is "operationalized" is critical to identifying the means and methods representing this potentially complex social phenomenon.^25^ Otherwise, a mismatch between meaning, value, and degree of "sustainability transformation" may lead to inequitable emphasis and intervention. For example, while sustainability is considered the "dynamic stability in social and ecological systems and their interactions," environmental sustainability is assessed differently based on "ecological footprint and ecosystem services".^25^ This mismatch leads to potential misunderstandings regarding the conceptual dimensions of sustainability and its assessment, especially when viewed from the different perspectives of ecology, politics, ethics, socioeconomics, democracy, culture, and theology.^26^

A search in the Scopus online database revealed several review papers addressing sustainable neighborhood assessment. Most articles focused on reviewing the existing NSA systems or a specific subtopic related to the NSA systems. For example, Sharifi et al.^20^^,^^27^ investigated the limitations and success factors of forty NSA tools from different parts of the world, respectively. Kamble and Bahadure^28^ examined twelve NSA tools from developed and developing countries to derive a framework for NSA in developing countries. Adewumi et al.^13^ analyzed four well-known NSA systems, including the LEED, BREEAM, Pearl, and Green Star tools using the Bellagio Sustainability Assessment Measurement Principles (STAMP). Tam et al.^29^ also reviewed 20 dominant NSA systems. Similarly, Borges et al.^21^ studied the two famous NSA tools, the LEED and the BREEAM, through the lens of cultural heritage. On the other hand, Cloutier et al.^30^ reviewed the relevant literature to capture the measures of happiness, economic, ecological, cultural, social, and sustainability for developing a new NSA tool promoting happiness in neighborhoods. The review of NSA in the French context by Chastenet et al.^31^ and a review of case studies assessing environmental impact through life cycle assessment (LSA) at the neighborhood level by Lotteau et al.^32^ are among the other review papers.

The existing review studies provide a considerable amount of knowledge and evidence about the limitations of the NSA tools,^13^^,^^20^^,^^21^ also on the improvement of the tools over the years.^27^ However, the existing knowledge and evidence are mainly related to well-known systems and tools, e.g., the LEED, the BREEAM, Green Star communities, etc. Therefore, more research is needed to have a complete picture of the NSA and to examine how the regular critique of the prominent tools has resulted in the improvement of sustainability assessment in neighborhoods.^27^

**Figure 1:**
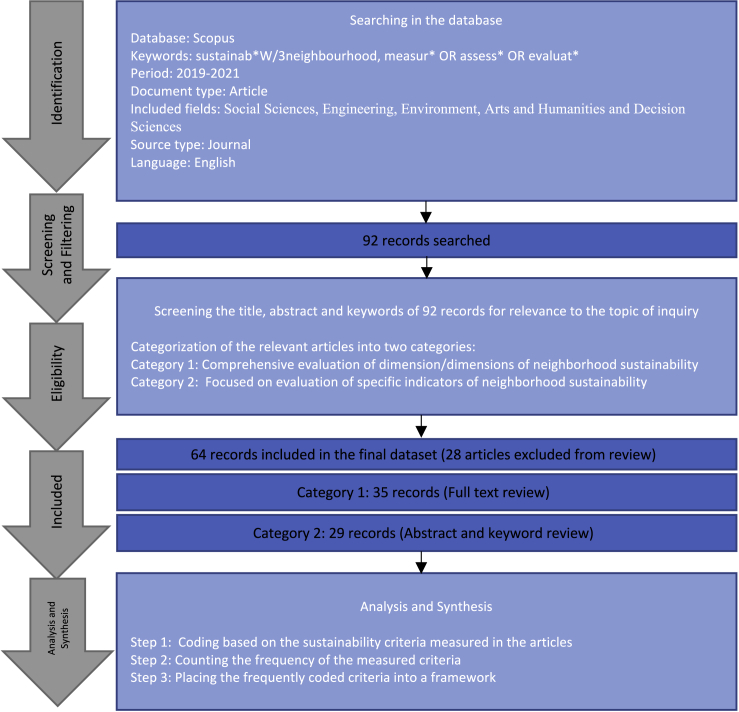
SLR methodology adopted in this study

As a result, this study builds on the existing knowledge of the NSA by focusing on empirical studies by researchers rather than analyzing the prominent NSA tools. The current research investigates how researchers, particularly academics, operationalize the concept of sustainable neighborhoods in their empirical studies, following the approach outlined by Salomaa and Juhola.^25^ The study approach will help look at the sustainable neighborhood concept from a different perspective. It will also provide an overview of how the extensive research and critique of the prominent neighborhood assessment tools have shaped the measurement of neighborhood sustainability in empirical studies by researchers. Consequently, a systematic literature review (SLR) of journal articles that featured sustainability measurement in neighborhoods, published between 2019 and November 2021, was subsequently performed. We analyzed 64 journal articles by categorizing them into category 1 (35) and category 2 (29) articles and recorded the frequency of the sustainability criteria measured in the papers. Figure 1 provides an overview of the study method.

Given this backdrop, the main aim of this study is to highlight the recent advancements in the sector of sustainable neighborhoods and create a better understanding of the sustainable neighborhood concept from the perspective of academicians. Specifically, we aim to answer the following two research questions.

1. What are the main factors contributing to the concept of a sustainable neighborhood in the recent empirical studies produced by academicians and researchers?
2. What is the relationship between the sustainable neighborhood criteria and how to integrate them into a framework?
Our findings suggest that form and morphology, community and sense of place, livability, equity, and viability are the main factors contributing to the sustainability of a neighborhood. Moreover, sustainable urban form and morphology are crucial factors affecting multiple dimensions of neighborhood sustainability. The following section explains the detailed study results, followed by the discussion and the limitations of the study. The STAR Methods sections provide the research methodology in detail.

## Results

**Figure 2:**
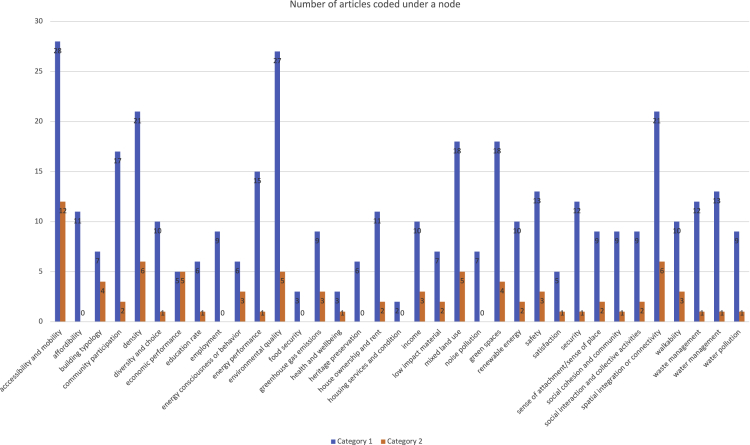
Frequency of the measured criteria in the two categories

Our results reveal that accessibility and mobility, environmental quality, spatial integration and connectivity, density, mixed land use, and green spaces are the most widely used criteria for sustainable neighborhood evaluation in category 1 of the retrieved articles, measured in at least 18 out of 35 papers (Figure 2). Category 2 also confirms the same results. In addition, accessibility and mobility is the most frequently measured criterion in category 2, appearing in 12 out of 29 articles. Figure 2 shows the number of articles in categories 1 and 2 that measure a neighborhood sustainability criterion, with blue and orange colors, respectively.

**Figure 3:**
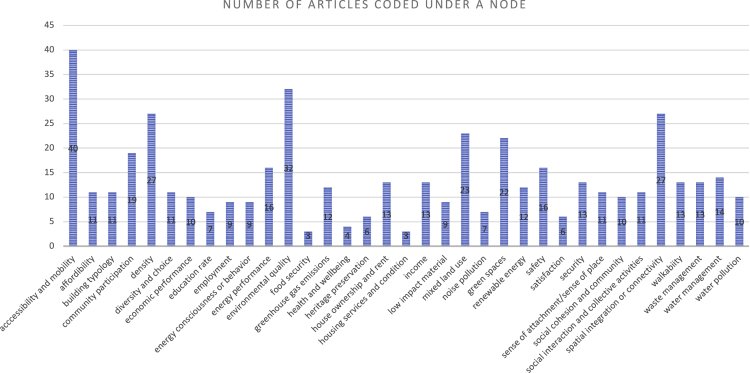
Overall frequency of the measured criteria

Consequently, as shown in Figure 3, out of a total of 64 analyzed articles both in category 1 and category 2, more than 15 papers (at least 30%) have measured aspects of accessibility and mobility, environmental quality, spatial integration and connectivity, density, mixed land use, green spaces, community and citizen participation, safety, and energy performance. In addition, 10%–30% of papers measured criteria related to house ownership and rent, water management, income, security, walkability, waste management, greenhouse gas (GHG) emissions, renewable energy, etc. Criteria including health and well-being, food security, and housing services and condition are the least appeared sustainable neighborhood criteria in the review papers.

**Figure 4:**
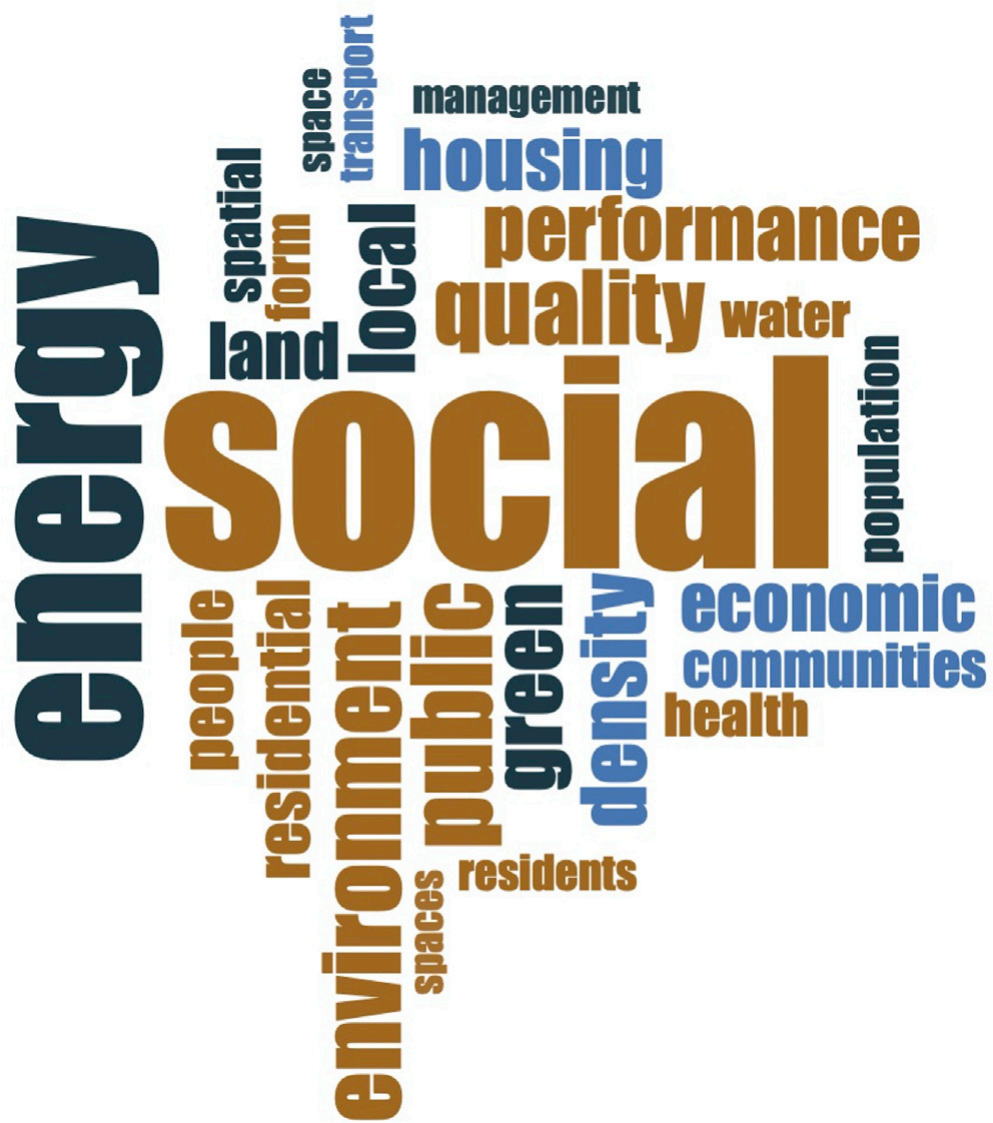
Word frequency (Category 1)

**Figure 5:**
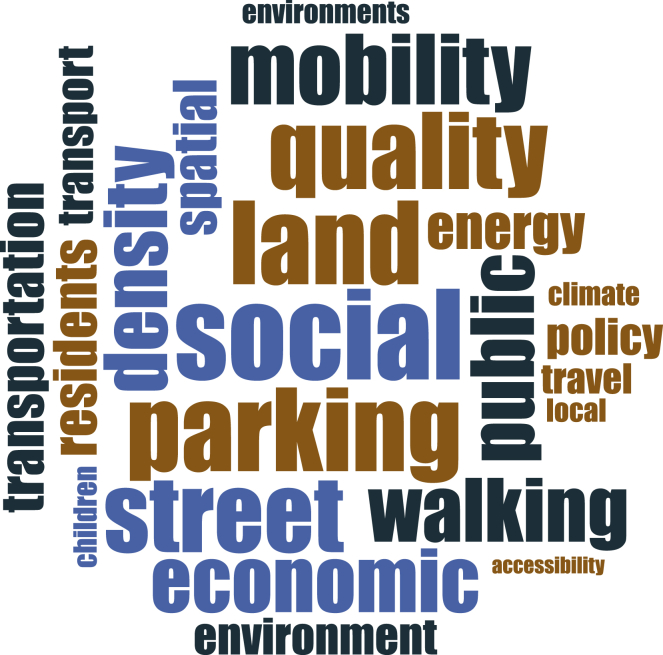
Word frequency query (Category 2)

To support our findings, we also performed a word frequency query for both categories of our data, i.e., the full text of the articles in category 1 and the abstracts of the articles in category 2. Figures 4 and 5 present the word frequency query (using NVIVO) for category 1 and category 2 of the papers, respectively. It is important to note that general terms, e.g., urban, sustainability, neighborhood, neighborhoods, sustainable, city, assessment, building, etc., were added to NVIVO’s stop words list for this query.

We found that "social" is the most frequently used term forming a weighted percentage of 0.77% and 0.60% in category 1 and category 2 of the papers, respectively. Energy (0.56%, 0.38%), quality (0.29%, 0.54%), public (0.31%, 0.48%), density (0.26%, 0.48%), and economic (0.23%, 0.48%) are among the other frequently used terms in both categories. The frequency of the words “social” and “public” indicates the importance of social sustainability and public participation in the sustainability of a neighborhood.^11^ Furthermore, it was observed that category 2 of articles (focused on measuring a single or few specific sustainability criteria) is inclined toward measuring the elements of sustainable transportation, as it is evident from the frequency of the terms transport, transportation, street, mobility, walking, etc.

### Factors contributing to a sustainable neighborhood

**Figure 6:**
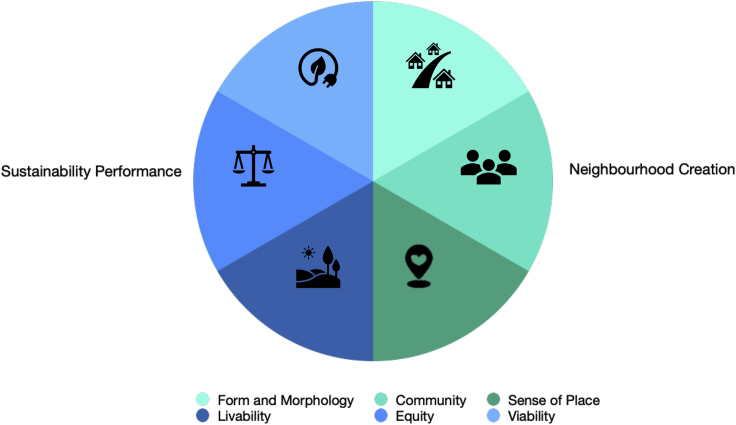
Factors contributing to a sustainable neighborhood

As explained in the STAR Methods section, the most frequently measured sustainability criteria (Figure 3) can be themed under the factors form and morphology, community, sense of place, livability, equity, and viability, based on the neighborhood definitions provided by Kallus and Law-yone^16^ and the sustainability framework defined by Tanguay et al.^33^ (See also Figures S1 and S2). These factors are further categorized into two main aspects of a sustainable neighborhood, i.e., the creation of a neighborhood and its sustainability outcome (performance). Table 1 and Figure 6 provide an image of the above discussion.

**Table 1:** Factors of a sustainable neighborhood

Category	Factor	Frequently measured Criteria (Figure 3)
Neighborhood Creation	Sustainable Form and Morphology (See Table 2 for details)	Environmental Quality, Density, Spatial Integration and Connectivity, Mixed Land Uses, Green Spaces, and Building Form and Typology
	Community	Community participation, Social interaction, and Social cohesion
	Sense of Place	Sense of attachment, Satisfaction, and Heritage preservation
Sustainability Outcome	Livability	Walkability, Environmental quality (Air Quality, Thermal Comfort, Lighting and Visual Comfort, Acoustic Comfort, Psychological comfort), GHG emissions, Waste management, Water management, and Water pollution
	Equity	Accessibility, Affordability, Safety, Security, Diversity and choice, Income rate, House ownership and rent, Employment rate, and Education level.
	Viability	Renewable energy, Energy-conscious or responsible behavior, and Economic performance (Creation of Agricultural green space, Installation of photovoltaic [PV] systems, and Installation of water harvesting systems)

**Figure 7:**
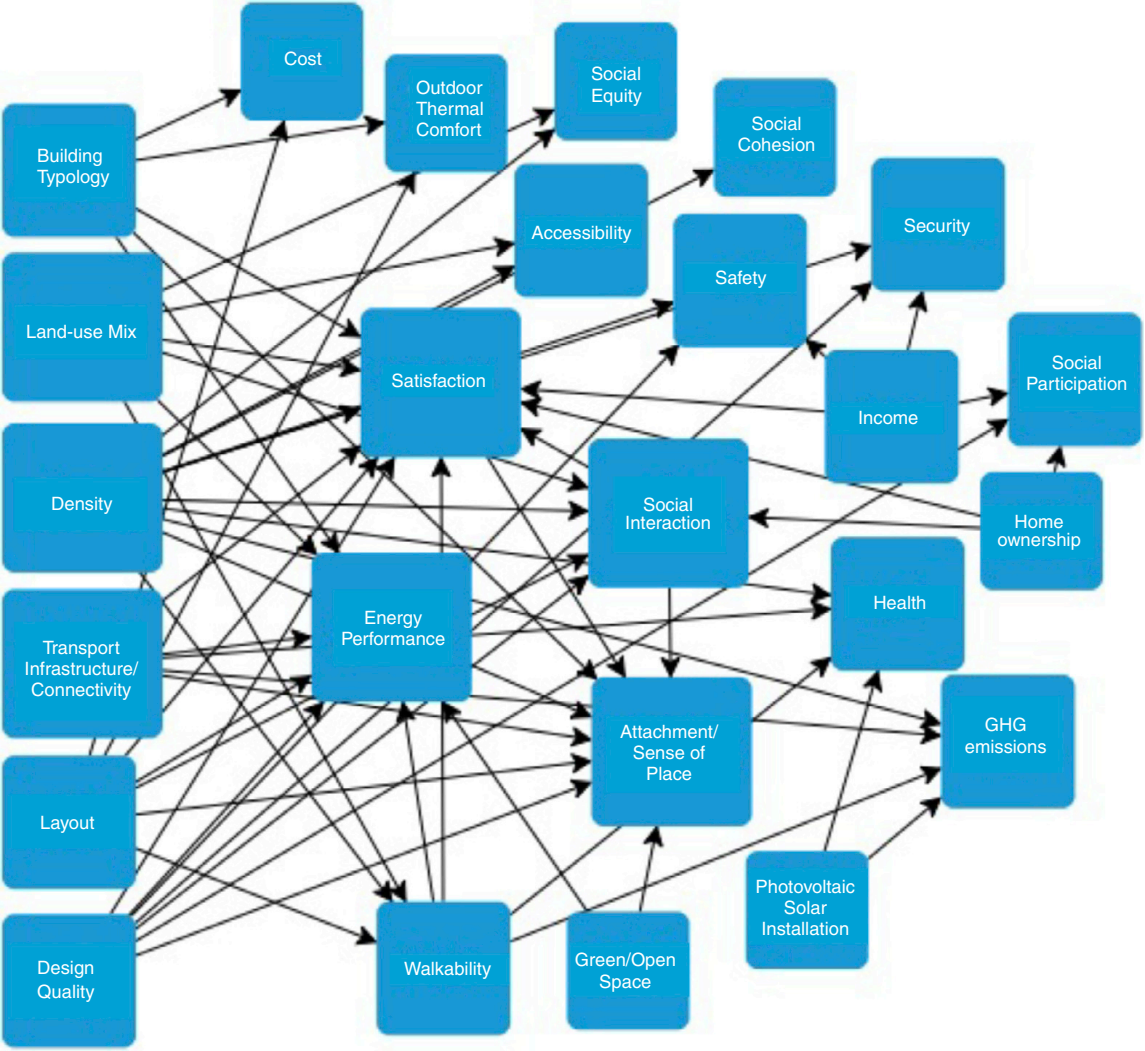
Relationship between urban form and morphology and the sustainability criteria measured in the reviewed articles^18^^,^^34^^,^^35^^,^^36^^,^^37^^,^^38^^,^^39^^,^^40^^,^^41^^,^^42^^,^^43^^,^^44^^,^^45^^,^^46^^,^^47^^,^^48^^,^^49^

Moreover, our review revealed that a neighborhood’s form and morphology affect sustainability in multiple dimensions, including economic, environmental, and social.^34^ Several of our reviewed articles studied the relationship between the physical form of neighborhoods and sustainability aspects. Researchers linked the neighborhood form and morphology to the quality of life,^35^^,^^36^ resident satisfaction,^37^ social sustainability,^38^^,^^39^^,^^40^^,^^41^ vitality,^18^ the socioeconomic composition of neighborhoods,^42^ environmental sustainability,^43^^,^^44^^,^^45^ solar energy access and resilience,^46^ sociocultural sustainability,^47^ and economic sustainability.^48^^,^^49^ Figure 7 visualizes the relationship between various sustainable neighborhood criteria from the reviewed articles.

**Figure 8:**
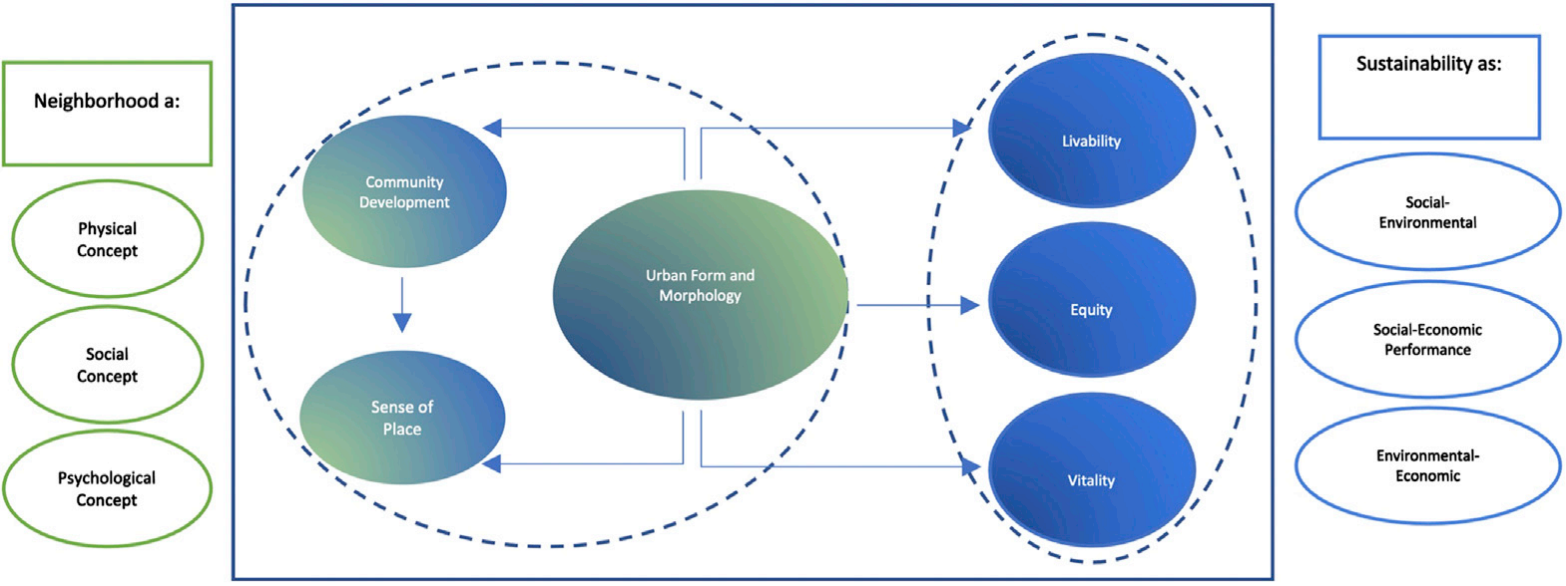
Relationship between urban form and morphology and other factors of a sustainable neighborhood

According to Figure 7, various sustainable form and morphology criteria such as density, land use mix, transport infrastructure/connectivity, and design quality affect social interaction, satisfaction and attachment (sense of place), energy performance, safety, security, and social equity. In light of the above information, it is safe to suggest that sustainable form and morphology as a factor impacts all the other sustainable neighborhood factors mentioned in Table 1 and Figure 6. We visualized the interrelationship between urban form and morphology and the other factors in Figure 8. It also emphasizes the importance of creating an efficient neighborhood with its three essential components (i.e., efficient form, community, and sense of place) to impact the social-environmental (livability), socio-economical (equity), and environmental-economic (vitality) performance of neighborhoods. The following subsections provide a brief introduction to all six factors.

#### Sustainable form and morphology

Urban form and morphology define the physical structure of a city, including building layout, land uses, green spaces, and road patterns. Based on our review, we suggest urban form and morphology as the primary factor affecting neighborhood sustainability.

It is noteworthy that the urban form is measurable at several scales, from buildings to blocks to cities. For example, mixed land use and street layout comprise the measures of urban form at a neighborhood scale, whereas density is measurable at both micro and macro scales.^39^ Likewise, Liu and Li^47^ categorized the neighborhood’s physical form into three groups of spatial characteristics, i.e., buildings, open spaces, and blocks (neighborhood), to study its impact on the sociocultural sustainability of a neighborhood in Beijing.

Although there exists no consensus on the most appropriate model for a sustainable neighborhood, based on our review, the widely used criteria for sustainable neighborhood form and morphology evaluation are environmental quality, density, spatial integration and connectivity, mixed-land use, green spaces, and building form and typology. Table 2 presents the measures of evaluating these criteria from our reviewed articles.

**Table 2:** Criteria defining sustainable urban form and morphology

Criteria	Measures	References
Environmental Quality	Passive and active solar design, UHI (urban heat island), Quality of open space in terms of material, equipment, shading devices, routes, and sidewalks design, public activity and leisure spaces, urban furniture (sitting benches), landscape elements, lighting, details, frontage, access to public spaces, Maintenance of buildings and open spaces, Esthetic appeal, Attractiveness, Cleanliness, Car-parking design	^5^^,^^10^^,^^11^^,^^37^^,^^38^^,^^39^^,^^40^^,^^41^^,^^43^^,^^45^^,^^48^^,^^49^^,^^50^^,^^51^^,^^52^^,^^53^^,^^54^^,^^55^
Density	Population Density (e.g. population/hectare, persons per acre, people per km2), Compactness (floor area ratio), Development Footprint or DF (developed land area per population), Built density (dwelling units per hectare, constructed area per surface of the neighborhood), Perceived Density (Perceived Crowdedness)	^10^^,^^15^^,^^17^^,^^36^^,^^37^^,^^38^^,^^39^^,^^41^^,^^44^^,^^45^^,^^47^^,^^51^^,^^56^
Spatial Integration and Connectivity	Road network, Road connectivity (the total length of road/area of the neighborhood), Spatial connectivity with adjacent neighborhoods, Distance to the city center, Spatial connectivity of the street network in neighborhoods (wide or high-speed peripheral streets), traffic levels, Density of street networks, Directness of paths, Street configuration and street hierarchy (types of roads/streets/paths in site); Intersection density in street network (intersections/square mile), other street network measures (characteristic path length, cyclomatic number, alpha index, beta index, gamma index), Road widening area, Road width, Sidewalk width, Length of pedestrian paths; Length of cycling paths, Dimensions of street blocks frontages, Buildings’ spatial relationships (Back-to-back/Side-to-side), Block/Plot’s connection to its immediate surrounding context by public roads, semipublic roads, and alleyways	^10^^,^^15^^,^^17^^,^^18^^,^^37^^,^^38^^,^^41^^,^^46^^,^^47^^,^^48^^,^^49^^,^^51^^,^^52^^,^^54^
Mixed Land Uses	Diversity in land use, i.e. provision of various housing types, services, facilities, amenities (clinics, schools, parks, sports centers, supermarkets, etc.), and open space through map analysis or field observation, Dissimilarity indices (entropy index and balance index), Percentage of commercial land, waterbody, industrial, institution, multi-family houses, single-family houses, recreation/parks, vacant or agricultural land, religious facilities, and roads, Residential land use per business land use ratio, Number of mixed-use plots, Residential per nonresidential area ratio, Economic floor area per total floor area ratio, Residential floor area per total floor area ratio, Single-function block area per neighborhood area ratio, Perceived land use mix (walkability to diverse functional land uses)	^17^^,^^18^^,^^35^^,^^37^^,^^38^^,^^39^^,^^42^^,^^43^^,^^50^^,^^51^^,^^52^^,^^54^
Green Spaces	Green area density (Green area per unit area ratio), Proximity or Distance to green areas, Green space change rate, Accessibility to green spaces, gardens, and parks, Protection and preservation of green areas, Entropy Index, View of green spaces, Trees (block, external, street), Green roofs, Green facades, Green spaces for urban agriculture, Number of landscape assets, Green areas design, Provision of public green spaces in neighborhoods	^2^^,^^5^^,^^10^^,^^15^^,^^42^^,^^43^^,^^48^^,^^49^^,^^50^^,^^52^^,^^53^
Building Form and Typology	Plot size and built proportion including types of external areas, such as side gardens, front gardens, parking lots, patios, etc., H/W ratio of the buildings, Building height (e.g., low-rise, high-rise, or number of stories), Construction type of building (e.g., brick, mix, mud), Building use/function, Age, Style (e.g., Victorian), Types of housing (courtyards, duplex, triplexes, and galleries/apartment or single-family houses, rowhouses, etc.), Housing shapes (e.g., L-shape)	^18^^,^^35^^,^^37^^,^^41^^,^^48^^,^^49^^,^^53^

#### Community development

Creating a community is another crucial factor in a sustainable neighborhood. The objective is to create a vibrant social community with active public participation.^57^ According to researchers, it is the presence of a community bond that holds a neighborhood together.^37^ Community participation is considered a critical component of social sustainability. It investigates residents’ engagement in community activities and volunteering to help improve their neighborhoods.^41^ Several types of community activities can be practiced in a neighborhood, including charity work, local elections, neighborhood-related projects, involvement in recreational facilities (i.e., sports, community center), and membership of community groups (i.e., sports teams, church groups). Several of our reviewed articles emphasize the importance of community participation in creating a sustainable neighborhood.^11^^,^^17^^,^^37^^,^^40^^,^^50^^,^^51^^,^^52^^,^^53^^,^^56^^,^^57^^,^^58^

Social interaction, however, works as a social glue to hold the community together.^39^ A socially sustainable neighborhood is where people live, work, and interact together. Lack of social interaction can significantly impact residents’ sense of attachment to their community.^40^ Human interaction in neighborhoods is believed to increase the feeling of safety and satisfaction between residents, contribute to social networks and quality of life, and promote social sustainability and economic development of the community.^18^^,^^38^^,^^39^^,^^54^

Social cohesion is another critical element in a community linked with social participation and social networks.^39^ Civic involvement and joint efforts from the key stakeholders in a community strengthen social cohesion.^57^ Social cohesion addresses the existence of strong community bonds formed based on social trust and support in the absence of social conflict in a community.^52^^,^^55^

Based on our review, the community and participation, social interaction, and social cohesion nodes occupied more than 35% and 20% of the reviewed articles. Therefore, we define this factor by community participation, social interaction, and social cohesion in communities.

#### Sense of place

As much as neighborhoods constitute a physical and social dimension, they also form a psychological concept.^16^^,^^59^ Sense of place defines the emotional bond between people and their neighborhoods, i.e., the feeling of belonging and attachment.^16^^,^^39^ Sense of place is interrelated to people’s satisfaction and enjoyment of their neighborhood. Researchers believe that the perception and satisfaction of inhabitants determine the level of social sustainability in an urban environment.^39^^,^^60^ People who feel attached to their neighborhood and have strong ties with their community are more likely to stay there and involve in their neighborhood’s improvement and continued development.^15^^,^^39^^,^^54^ Several urban form factors and socioeconomic features are linked to affect the sense of place in residents of a neighborhood.^39^^,^^61^ Consequently, we define this factor by the criteria sense of attachment, satisfaction, and heritage preservation, resting on our review.

#### Livability

Livability constitutes varying definitions and indicators. For example, some of the indicators defining livability are convenient transportation, proximity to basic amenities, health, safety, affordability, environmental quality, social engagement, and economic and educational opportunities.^62^^,^^63^^,^^64^

However, according to many, livability is synonymous with quality of life. Consequently, for a place to be livable, it should provide a livelihood to its inhabitants and preserve the environment.^62^ This definition is similar to Tanguay et al.’s^33^ definition of livability: the interaction between the social and environmental sustainability dimensions. Following this definition, we placed walkability, environmental quality (air quality, thermal comfort, lighting and visual comfort, acoustic comfort, and psychological comfort), GHG emissions, waste management, water management, and water pollution criteria into this factor.

#### Equity

Equity defines the interaction between social and economic dimensions of sustainable development.^33^^,^^65^ Equity has become a common research theme as the Agenda 2030 for Sustainable Development emphasizes leaving no one behind.^65^ Researchers define various criteria to address equity in a neighborhood, such as job accessibility, accessibility to public services and green spaces by walking or public transit, and affordable housing.^65^^,^^66^^,^^67^^,^^68^^,^^69^^,^^70^^,^^71^^,^^72^ However, based on our reviewed papers, equity is defined by accessibility, affordability, safety, security, diversity and choice, income rate, house ownership and rent, employment rate, and education level.

#### Viability

The interaction between the economic and environmental sustainability dimensions defines the concept of viability. Thus, economic progress supporting the ecosystem capacity and avoiding the depletion of renewable resources is considered viable.^33^ Consequently, we themed renewable energy, energy-conscious or responsible behavior, and economic performance (i.e., economic evaluation of environmental solutions such as the creation of agricultural green space, installation of photovoltaic (PV) systems, and installation of water harvesting systems^2^^,^^73^) under this factor.

## Discussion

There is plenty of research on assessment tools and indicators of a sustainable neighborhood. Besides, new tools are constantly developing for specific contexts. Consequently, a considerable amount of research focuses on the characteristics and shortcomings of the established NSA tools. However, in this article, we aimed to understand the concept of a sustainable neighborhood from the perspective of researchers and to identify the main factors that contribute to neighborhood sustainability through their empirical studies.

Our reviewed articles suggest that physical form and morphology are the most crucial factors for defining a sustainable neighborhood. Sustainable form and morphology are associated with creating community, sense of place, equity, livability, and viability, which are the other crucial factors of neighborhood sustainability found in this research (Figure 8).

The study also highlights how researchers have responded to the current criticism of prominent NSA tools and their proposed solutions for better assessment. For example, the lack of consideration of interlinkages between sustainability aspects and the dominant focus on environmental sustainability are among the debated limitations of the NSA tools.^11^^,^^20^ Nonetheless, in our study, the review papers have used tools and frameworks to assess the interlinkage between urban form and social sustainability,^38^^,^^39^ spatial characteristics and sociocultural sustainability,^47^ spatial assessment and multiple sustainability dimensions,^5^ and urban form and the triple bottom line of sustainability.^37^ Refer to Figure 7.

Non-transparent and top-down approaches adopted in the NSA systems are another debated limitation of these systems.^20^ The reviewed literature responds to it by supporting public participation and promoting various bottom-up methods, including analyzing citizens’ opinions on urban sustainability,^53^ assessing people’s perception and satisfaction with their neighborhoods,^37^^,^^39^^,^^52^ geospatial assessment,^5^ developing ICT (Information and Communication Technology)and mobile apps to support community-based interventions,^57^^,^^56^ and adopting participatory approaches for indicator development.^50^

In addition, the limitation of context-specific issues is another limitation; a few studies responded to this matter by using Delphi methods and multi-criteria analysis to find context-specific sustainability indicators.^11^^,^^17^^,^^50^

The above discussion and the review findings indicate that social sustainability is vital for sustainable neighborhood development and thus forms an essential part of the NSA. While this is a prime limitation of the NSA tools, our review provides various criteria to assess social sustainability. Despite the higher frequency of environmental indicators in the reviewed papers, it is noteworthy that researchers integrated environmental assessment with social approaches, e.g., by assessing public opinion about environmental problems. Thus, it conforms with the newer conception of sustainability that promotes people-oriented and participative attitudes to address environmental issues.^74^^,^^75^

Another relevant issue is that research on this topic comes from various countries; yet, the European Union (EU), the UK, China (including Hong Kong), and the US are the main areas contributing to the reviewed papers. Thus, this may overshadow the sustainability issues in economically less stable countries. For example, the criteria “housing services and condition” (satisfactory living conditions and having basic facilities such as electricity and piped water system, etc.) is only mentioned in three papers, which might be a prominent issue in developing countries (Figure 3). Owing to plenty of articles from the developed regions and their impact on the research in developing countries, these criteria remain hidden.

Moreover, the review finds that sustainable transport, sustainable energy, and GHG emissions seem to be the primary concern of EU countries (specifically, Germany, Belgium, and Italy) when addressing neighborhood sustainability. However, the UK and the US show a significant interest in aspects of social sustainability, e.g., gentrification (equity), happiness, and values.

### Conclusion

As sustainability assessment and monitoring have become the main focus of policymakers and researchers to understand the means and methods representing the complex concept of sustainability in cities, we believe our paper has multiple contributions. It contributes to expanding the existing knowledge on neighborhood sustainability evaluation, adds to the literature on designing and monitoring sustainable cities and communities, and contributes to achieving SDG 11. The paper provides valuable information for scholars and practitioners in the field; it gives an insight into the widely measured criteria by researchers and thus highlights the overlooked factors which need inclusion in the NSA methods and tools. Moreover, unlike many other articles, we discussed the idea of sustainable neighborhood measures in the light of the neighborhood definitions, i.e., neighborhood as a physical, social, and psychological concept (Figure 8), which we believe is central for addressing neighborhood sustainability. However, in approval of the previous literature, the review proposes the need to develop more local or context-based sustainable neighborhood frameworks and assessment criteria.^15^^,^^50^^,^^52^

### Limitations of the study

As with every research, our article also has its limitations. We based our review only on empirical articles, whereas future research can also include theoretical papers that address neighborhood sustainability assessment. Moreover, we limited the publication period from 2019 to 2021, while future researchers can extend the time to cover more articles. We suggest expanding the search period by using the search string, as used in this article, and including the more recent articles published in 2022 and afterward to better study the geographic differences of the topic. As the Asian and African regions are witnessing a boom in NSA systems,^34^^,^^50^ it will help to identify how sustainable neighborhood development is different in developing and developed countries. Expanding the study period to include papers published after 2015 would also be beneficial. It would help unearth the impact of the United Nations Agenda 2030, particularly SDG 11, on the sustainability evaluation of neighborhoods. It will also provide plenty of papers to highlight the geographic differences in worldwide sustainability measures.

## STAR★Methods

### Key resources table

**Table:** 

REAGENT or RESOURCE	SOURCE	IDENTIFIER
**Software and algorithms**		

NVIVO 12.6.0	QSR International	https://www.qsrinternational.com/nvivo-qualitative-data-analysis-software/home

**Other**		

Journal Articles (Table S1)	Scopus	www.scopus.com

### Resource availability

#### Lead contact

Further information and requests for resources should be directed to and will be fulfilled by the lead contact, Mahsa Khatibi (mahsa.khatibi@yahoo.com).

#### Materials availability

The study did not generate new unique materials.

### Method details

The systematic literature review (SLR) approach has become a popular tool for depicting the rapid development of disciplines.^25^ It aims to answer specific questions by gathering empirical evidence through clear and systematic methods from qualified studies.^76^ Based on the steps proposed by Salomaa and Juhola,^25^ Kong et al.,^76^ Det and Hallinger,^77^ and Tanguay et al.,^33^ this study used a three-step process for performing the review: 1). Identification of literature, 2). Screening and filtering the literature for relevant papers, 3). Analysis and synthesis of the literature. (Refer to Figure 1).

#### Identification of the literature

The first step includes defining a search protocol and searching in relevant databases. Consequently, we looked up the Scopus database using the keywords “sustainab∗ W/3 neighborhood” and “measur∗ OR assess∗ OR evaluat∗” in titles, abstracts, and keywords of the articles. The search terms were selected to find the possible combinations of sustainability and neighborhood (such as sustainable neighborhood, sustainable urban neighborhood, and sustainability in the urban neighborhood, etc.) and its measurement, assessment, or evaluation. We used the Scopus database due to its broader coverage of research areas compared to the Web of Science.^27^

The mentioned keywords with other applied Scopus filters, explained below, yielded 198 papers in April 2021.

According to the review paper by Grazieschi et al.,^78^ a sustainable neighborhood is a dynamic concept evolving with time. Therefore, it is relevant to study the state-of-art of sustainable neighborhood concept. Moreover, as mentioned in the introduction section, previous review papers have either focused on studying the NSA tools or a broad overview of the sustainable neighborhood concept due to selecting a wide-ranging publication period.

Consequently, to deeply examine the state-of-the-art sustainable neighborhood criteria, we selected the publication period of the search string, described above, between 2019 and 2021 to limit the number of papers. As a result of the constantly evolving definitions of a neighborhood and sustainable neighborhoods,^16^^,^^78^ we found it reasonable to capture the new knowledge by limiting the search period from 2019 onwards. It is also valuable as it will reveal if the extensive critique of the prominent NSA tools shaped the present-time measurement of neighborhood sustainability in researchers’ empirical studies. To further justify our approach, we performed multiple rounds of searches from April to November to track and include the articles newly added to the Scopus database. Finally, the last search round with the search string, as explained earlier, performed on November 23, 2021, and a publication period limited from 2019 to 2021, retrieved 92 papers. We then used these 92 papers for the second step of the review process, i.e., screening and filtering the searched literature for relevance to the subject of study.

#### Screening and filtering the literature

In the second step, we screened the abstract, title, and keywords of the 92 articles for relevance to our subject of inquiry. As a result, we filtered the 92 articles into three categories. The first category involves papers that evaluated at least one dimension of sustainability (i.e., social, environmental, economic) through multiple indicators or studied the relationship between a sustainable neighborhood criterion and the neighborhood’s overall sustainability through a comprehensive framework. We downloaded this category for full-text reading and further analysis.

Consequently, the second category of papers includes articles that evaluate a single criterion or a narrow topic related to some aspect of neighborhood sustainability. We reviewed the articles in this category only through their abstracts and keywords to identify the sustainability criteria measured in them; we downloaded the full text of the article only when we felt it was necessary to understanding the content.

Finally, the third category comprises the excluded articles due to subject irrelevance after screening their abstracts, titles, and keywords. The excluded articles included papers missing the term neighborhood or articles discussing or proposing neighborhood sustainability assessment frameworks without an empirical application on a case study.

For example, the research paper entitled “Social sustainability of compact neighborhoods evidence from London and Berlin”^41^ uses a holistic framework to measure social sustainability in case studies from London and Berlin. Thus, we placed it in the first category of review papers for reading its full text to identify the social sustainability criteria measured in it. On the other hand, the article entitled “Bottom-up strategies for shared mobility and practices in urban housing to improve sustainable planning” was placed in the second category and carefully read through its abstract and keywords to identify the mobility criteria measured in it. However, the article entitled “The quest for an adequate test: Justifying the sustainable city as an order of worth” was placed in the third category of articles for exclusion after screening its abstract, title, and keywords due to its theoretical nature.

Consequently, this step led to placing 35 articles in the first category for full-text reviewing and 29 papers in the second category to identify the sustainability criteria through their abstracts and titles, whereas 28 fell into the third category for exclusion from the review process. The 64 articles included in the literature review are listed in the Table S1.

#### Analysis and synthesis of the literature

The primary approach used in this paper is the frequency of criteria.^23^^,^^33^ NVIVO version 12.6.0 and Microsoft Excel are the tools used for analysis purposes. NVIVO is a software program used in qualitative research methods for simplifying coding processes and data analysis.^79^

At first, we added the full text and the abstracts of the articles in the first and second categories, respectively, to NVIVO. The added papers were then manually reviewed to identify the sustainability criteria measured in them. For each sustainability criterion, we created a node in NVIVO. A node represents a code that collects and stores references about a specific theme or relationship. Every sustainability criterion measured in the papers was then coded under its respective node, i.e., the text describing a criterion in the articles was selected and referenced under a node. The appropriate node is selected from the list of the already created nodes by using the “Code Selection” option under the “Code” command in the menu bar. It is noteworthy that, during the coding process, as new knowledge developed, we refined the nodes several times. Once the coding process was complete, we summed the number of articles referenced under a node, i.e., the total number of papers measuring a single criterion, to determine its frequency. We used Microsoft Excel for the graphical representation of the data, such as in Figures 2 and 3. Moreover, a separate node called “relationship” was also created to code the discussed or measured relationships between the sustainability criteria in the articles.

Finally, the criteria measured in more than 10% of the articles (i.e., occurring in at least 6 of the reviewed papers) were themed into factors to form a conceptual framework. According to Tanguay et al.,^33^ based on Niemeijer and de Groot,^80^ placing the obtained criteria into a conceptual framework is a crucial step in ensuring that they (selected criteria) accurately reflect the study phenomena. There are no precise models to simplify the interactions that determine sustainable development, and the obtained indicators can be organized into various possible approaches. However, to capture the intricacy of sustainable development, frameworks that focus on at least two of the following issues, such as objectives, domains, challenges, sustainable development elements, and cause and effect relations, can be beneficial.^33^ In light of the above knowledge, drawing on theories and predetermined definitions of the neighborhood and sustainability, we iteratively developed our final framework as it is shaped and refined by findings from the reviewed papers.

According to Kallus and Law-yone,^16^ a neighborhood is a place that creates a social connection between people, provides efficient physical features, and causes a bond between the people and their home (Figure S1). Besides that, Tanguay et al.^33^ frame the interactions between social, environmental, and economic dimensions of sustainability by defining livability, equity, and viability, as shown in Figure S2. Consequently, we themed the sustainability criteria occupying more than 10% of the reviewed papers under the factors of form and morphology, community, sense of place, livability, equity, and viability (Figure 6).

## Data Availability

• This paper analyzes existing, publicly available articles from the Scopus database. The reviewed articles are listed in the Table S1. • NVIVO is used for analysis and the file is available from the lead contact upon reasonable request. • Any additional information required to reanalyze the data reported in this paper is available from the lead contact upon reasonable request.

## Supplemental information

Document S1. Figures S1 and S2

Table S1. List of the reviewed journal articles, related to the key resources table (KRT)
