# Supplementary material for: Measuring the sustainability of neighborhoods: A systematic literature review
Source: iScience. 2023 Jan 10;26(2):105951. doi: 10.1016/j.isci.2023.105951 (PMC9971876; doi:10.1016/j.isci.2023.105951)
Supplement: Document S1. Figures S1 and S2 [file mmc1.pdf]

**iScience, Volume *26***

## Supplemental information

# Measuring the sustainability of neighborhoods: A systematic literature review

**Mahsa Khatibi, Khairul Anwar Mohamed Khaidzir, and Sharifah Salwa Syed Mahdzar**

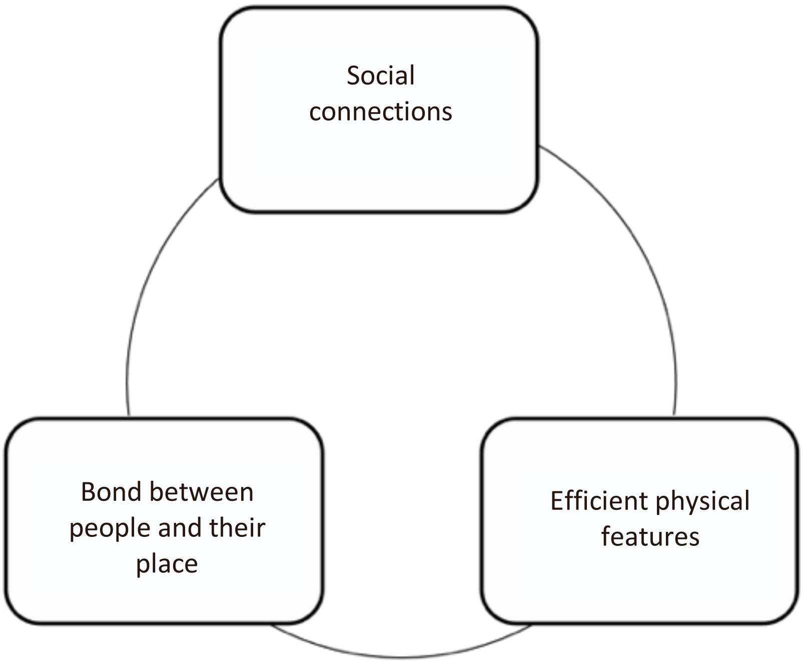


Figure S1. Neighborhood Definition [1], related to the Method details section in STAR Methods, Figure 6, and Figure 8

2

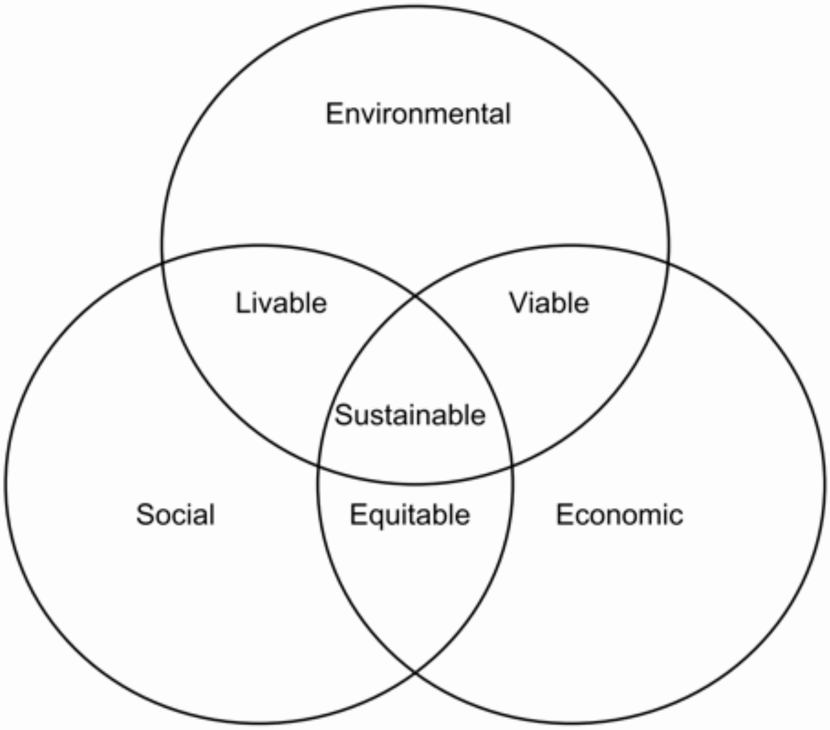


Figure S2. Dimensions of Sustainability[2], related to the Method details section in STAR Methods, Figure 6, and Figure 8

REFERENCES:

1. Kallus, R., and Law-yone, H. (2000). What is a neighbourhood ? The structure and function of an idea. Environ. Plan. B Plan. Des. *27*, 815–826. 10.1068/b2636.
2. Tanguay, G.A., Rajaonson, J., Lefebvre, J. ean-F., and Lanoie, P. (2010). Measuring the sustainability of cities : An analysis of the use of local indicators. Ecol. Indic. *10*, 407–418. 10.1016/j.ecolind.2009.07.013.
